# Maternal Near Miss Morbidity and Mortality: An Audit Analysis of 10 Years From a Private Tertiary Care Obstetric Center

**DOI:** 10.7759/cureus.82031

**Published:** 2025-04-10

**Authors:** Pallavi Chandra Ravula, Anisha Gala Shah, Sailaja Devi Kallur, Nuzhat Aziz, Tarakeswari Surapaneni, Ananta Ghimire

**Affiliations:** 1 Obstetrics and Gynecology, Fernandez Foundation, Hyderabad, IND; 2 Obstetrics and Gynecology, Fernandez Hospital, Hyderabad, IND; 3 Biostatistics, coGuide Academy, Bangalore, IND

**Keywords:** hospital data audit, maternal mortality, maternal near miss morbidity, potentially life-threatening conditions, quality of obstetric care, record-based analysis

## Abstract

Background

Maternal near-miss morbidity (MNM) and maternal death (MD) are indicators of the quality of maternal health care. Despite various efforts, MNM and MD rates remain high in several nations. Therefore, it is important to develop new strategies to address this issue.

Objectives

The primary objective of this study was to determine the proportion of potentially life-threatening conditions (PLTC), life-threatening conditions (LTC), MNM cases, and MD cases among all women booked for care at the study site over the last 10 years. The secondary objectives of the study were to calculate the maternal morbidity and mortality indices and elucidate the underlying profile of MNM and MD cases.

Materials and methods

A clinical audit of data from Fernandez Hospital, Hyderabad, India, from January 2011 to December 2020 was carried out. Data of women diagnosed with PLTC during the study period were extracted. Incidences of MNM and MD were calculated along with other mortality and morbidity indicators. Etiologies of MNM and MD were evaluated.

Results

Of the 79,069 women included in the study, PLTC, LTC, MNM, and MD were noted in 7410 (9.37%), 245 (0.31%), 233 (0.29%), and 12 (0.015%) cases, respectively. Hemorrhage and preeclampsia were seen in 88 (37.77%) and 44 (18.88%) cases, respectively. Hypertensive disorders were the most common cause of MD, as seen in five (41.7%) cases.

Conclusions

Hemorrhage and hypertension were the leading causes of MNM in our study, and hypertension was the leading cause of MD. It is crucial for maternity centers to implement strategies to identify at-risk women. Such cases should be evaluated, diagnosed, and treated as soon as possible to avoid MNM and preventable MD.

## Introduction

The International Federation of Gynecology and Obstetrics and the World Health Organization (WHO) define maternal near miss (MNM) as women who barely survive death due to complications during pregnancy, childbirth, or within 42 days of termination of pregnancy [1,2]. The majority of cases of maternal morbidity and mortality are preventable, and timely identification and appropriate treatment of morbid conditions often prevent mortality [3]. Maternal morbidity and maternal death (MD) can serve as indicators of healthcare quality [4]. Approximately 800 mothers die each day due to causes that can be prevented through appropriate management. However, the global maternal mortality rate (MMR) decreased by 34% from 2000 to 2020, with 95% of deaths attributed to preventable causes [5]. In 2020, low- and high-income countries exhibited MMRs of 430 and 12 per live 100,000 births, respectively, with this difference highlighting a gap in medical care for low-income countries.

The United Nation’s Sustainable Development Goals aim to achieve an MMR of <70 by 2030 [6]. MNM has reported incidence of 15.9, 7.8, and 9.6 per 1,000 live births in lower-middle, upper-middle, and middle-income countries, respectively [7]. In 2022, India’s Sample Registration System released a maternal mortality bulletin reporting an MMR of 97 per 100,000 live births for 2018-2020, representing a 33% decrease compared to 2014-2016 data, with the states of Madhya Pradesh and Kerala having the highest (15.3%) and lowest (0.9%) MMR, respectively [8].

During pregnancy, labor and puerperium women can experience potentially life-threatening conditions (PLTC) with possible outcomes including MNM, MM, or maternal morbidities, which may manifest early or persist as sequelae (e.g., uterine prolapse and urinary incontinence) [9]. In 2011, WHO proposed a three-step guideline for standardized identification of MNM comprising baseline assessment, situational analysis, and intervention to improve the quality of obstetric care [9,10].

In 2014, India’s Ministry of Health and Family Welfare adopted the above-mentioned WHO guidelines [11]. For monitoring the quality of obstetric care, four MNM indicators were established: MNM incidence ratio (i.e., number of MNM cases per 1,000 live births), MNM:MD ratio, Mortality Index (MI, i.e., number of maternal deaths divided by the number of women with life-threatening conditions) expressed as a percentage [MI = MD/(MNM + MD)], and severe maternal outcome ratio (SMOR) [10].

Several studies have been carried out to determine the MNM, MM, morbidity indices, and associated factors in the Indian population [12]. However, few audits were carried out with study durations of >5 years. Therefore, the current study was carried out with the primary objective being to determine the rates of PLTC, LTC, MNM, and MD among all women treated at Fernandez Hospital, Hyderabad, India, over a study duration of 10 years. The secondary objectives of the study were to calculate the maternal morbidity and mortality indices among the study population, and to elucidate the underlying profile of MNM and MM cases.

## Materials and methods

This record-based audit was carried out at Fernandez Hospital, Hyderabad, India, which is a private tertiary care obstetric center, with approximately 9,000 births per annum. The study site initiated a criteria-based quality of obstetric care audit in 2010, using the WHO criteria [10]. This study is based on the data collected from 01 January 2011 to 31 December 2020. The study was approved by the Institutional Review Board (IEC Ref. No.: 01_2021) and conducted in accordance with the 1964 Helsinki Declaration and its later amendments or comparable ethical standards.

Using WHO near-miss criteria, the study site implemented a criteria-based quality of obstetric care audit in 2010 to evaluate an evidence-based modification of the protocols for ensuring the quality of obstetric care. Data of all the women registered for care and admitted to the hospital during pregnancy were extracted from the hospital medical records database. For each case, all clinical, management, and lab parameters were collected as per the WHO clinical criteria. Details related to follow-up for 42 days post-birth were also extracted. In cases of transfer to another center for medical or personal reasons, follow-up data were also obtained to the extent possible until six weeks post-labor.

All morbidity and mortality predictors were noted, including maternal age, body mass index, gestational age at the time of birth, parity, singleton or multifetal pregnancy, mode of birth, medical disorders, and PLTC at the time of admission. All the collected data were entered into a Microsoft Excel spreadsheet, and RStudio Desktop Version 2022.07.0+548 was used for statistical analysis [13]. Prevalence of MNM and MM, MNM incidence ratio, MNM:MD ratio, MI, and SMOR were calculated. Descriptive analysis was carried out, with quantitative variables reported as mean and standard deviation, and categorical variables reported as frequency and percentage. Data were also represented using appropriate tables, graphs and pie chart.

The strategies that were implemented for improving patient outcomes at the study site during the study period were monthly audits and meetings, aggressive correction of anemia, implementation of the Maternal Early Warning Trigger (MEWT) tool, and intervention by the morbidly adherent placenta (MAP) team.

Monthly audits and meetings

Meetings were conducted with the entire clinical team, including doctors and nurses, for all cases of MNM and MD, and learning points were shared. Based on these learning points, protocols and practices were updated through discussion.

Aggressive correction of anemia

Anemia represents a correctable PLTC. More than 50% of Indian women are anemic. Hemogram testing was done at the booking visit for every woman and repeated in every trimester. Routine screening for hemoglobinopathies was done for every patient at the booking visit. Anemia was treated aggressively in the antenatal period. Dietary modifications, and usage of therapeutic doses of oral iron and intravenous iron if there was no response to oral iron, were included in the protocol for antenatal care as needed.

Maternal early warning trigger tool

The MEWT tool was designed to identify clinical deterioration and provide a pathway for management. The tool addresses the four most common causes of maternal morbidity and mortality: hemorrhage, sepsis, hypertension, and cardiovascular dysfunction. The MEWT tool, implemented at the study site since 2017, has helped in case management using the alerts indicated in the Maternal Early Obstetric Warning System chart. Using these four pathways has helped to recognize and manage PLTC early [14].

Morbidly adherent placenta (MAP) team intervention

The overall increase in the caesarean section rates globally has also led to an increase in MAP cases. MAP is associated with massive hemorrhage and the need for blood transfusions, and women with MAP can eventually develop PLTC and MNM. Therefore, women with MAP were referred to the MAP team, which monitored them throughout their pregnancy. The MAP team, consisting of doctors and nurses with the most surgical expertise, was formed to address the increase in MAP cases. The team was available around the clock (i.e., 24/7). The MAP team ensured that these women and their families were counselled regarding anticipated morbidities, and to prepare them for receiving any necessary support. These patients were educated about warning signs and symptoms that would require seeking emergency medical care. The hospital administration personnel helped in this endeavor by ensuring timely logistical support of procuring blood and blood products, which made it possible to achieve a lower MD rate despite greater PLTC rates.

## Results

The dataset included a total of 79,069 women over the study period, with a mean age of 28.04±4.11 years. During the study period, the incidence of PLTC was 9.37% (n=7,410), and that of LTC was 0.31% (n=245). There were 233 (0.29%) cases of MNM and 12 (0.015%) cases of MD, as shown in Table 1. The SMOR was 3.10 per 1,000 live births, the maternal near miss incidence ratio was 2.95 per 1,000 live births, the MNM:MD ratio was 19.4:1, and MI was 4.9% as shown in Table 2.

**Table 1:** Descriptive analysis of the study population (N=79,069)

Diagnoses	Frequency (%)
Potentially life-threatening conditions	7,410 (9.37%)
Life-threatening conditions	245 (0.31%)
Maternal near misses	233 (0.29%)
Maternal deaths	12 (0.015%)

**Table 2:** Morbidity and mortality indices in the study population.

Indices	Value
Severe maternal outcome ratio	3.10 per 1,000 live births
Maternal near miss incidence ratio	2.95 per 1,000 live births
Maternal near miss: maternal death ratio	19.4:1
Mortality index	4.90%

Table 3 provides a year-wise distribution of PLTC, MNM, and MD for the study duration. The proportion of PLTC was highest in 2020 (n=9,433; 11.0%) and lowest in 2011 (n=5,583; 4.32%), and that of MNM was highest in 2016 (n=34; 0.42%) and lowest in 2011 (n=8; 0.14%). There were three deaths each in 2014 and 2019, two deaths each in 2015 and 2018, and one death each in 2017 and 2020.

**Table 3:** Year-wise distribution of PLTC, LTC, MNM, and MD. PLTC: Potentially life-threatening conditions, MNM: Maternal near miss, MD: Maternal death.

Year	Total N=79,069	PLTC N=7,410 (9.37%)	LTC N=245 (0.31%)	MNM N=233 (0.29%)	MD N=12 (0.02%)
2011	5,583	241 (4.32%)	8 (0.14%)	8 (0.14%)	0 (0.00%)
2012	6,561	617 (9.40%)	15 (0.23%)	15 (0.23%)	0 (0.00%)
2013	7,175	702 (9.78%)	18 (0.25%)	18 (0.25%)	0 (0.00%)
2014	7,767	678 (8.73%)	24 (0.31%)	21 (0.27%)	3 (0.04%)
2015	7,473	623 (8.34%)	20 (0.27%)	18 (0.24%)	2 (0.03%)
2016	8,052	774 (9.61%)	34 (0.42%)	34 (0.42%)	0 (0.00%)
2017	8,331	834 (10.01%)	32 (0.38%)	31 (0.37%)	1 (0.01%)
2018	8,811	936 (10.62%)	36 (0.41%)	34 (0.39%)	2 (0.02%)
2019	9,883	967 (9.78%)	34 (0.34%)	31 (0.31%)	3 (0.03%)
2020	9,433	1,038 (11.0%)	24 (0.25%)	23 (0.24%)	1 (0.01%)
Total	79,069	7,410 (9.37%)	245 (0.31%)	233 (0.29%)	12 (0.02%)

In terms of etiology, hemorrhage was the leading cause of MNM cases (37.77%), followed by preeclampsia (18.88%) and infection (14.59%), as seen in Table 4.

**Table 4:** Various etiological characteristics seen among MNM cases. PPH: Postpartum hemorrhage, AKI: Acute kidney injury, HELLP: Hemolysis, Elevated liver enzymes and Low platelet count, DIC: Disseminated intravascular coagulation, H1N1: Swine flu influenza, LRTI: Lower respiratory tract infection, COVID: Coronavirus disease, ITP: Idiopathic thrombocytopenic purpura, SLE: Systemic lupus erythematosus, PPCM: Peripartum cardiomyopathy, CHD: Congenital heart disease, HOCM: Hypertrophic obstructive cardiomyopathy, CCF: Congestive cardiac failure, PAH: Pulmonary arterial hypertension, RHF: Right heart failure, RHD: Rheumatic heart disease, SVT: Supraventricular tachycardia, AF: Atrial fibrillation, TOF: Tetralogy of Fallot, CHF: Congestive heart failure, WPW: Wolff-Parkinson-White syndrome, Portal HTN: Portal hypertension, AFLP: Acute fatty liver of pregnancy

Etiological factor for MNM	Frequency (%)
Hemorrhage	88 (37.77%)
Adherent placenta	39 (16.74%)
PPH	35 (15.02%)
Post-surgical bleed	5 (2.15%)
Rupture uterus	5 (2.15%)
Post miscarriage	3 (1.29%)
Postsurgical bleed	1 (0.43%)
Preeclampsia	44 (18.88%)
HELLP	25 (10.73%)
Abruption	7 (3.00%)
Pulmonary edema	6 (2.58%)
AKI	2 (0.86%)
DIC	1 (0.43%)
Hypertensive crisis	1 (0.43%)
Hypertensive encephalopathy	1 (0.43%)
Liver hematoma	1 (0.43%)
Infections	34 (14.59%)
Dengue	19 (8.15%)
H1N1	3 (1.29%)
Hepatitis E	3 (1.29%)
LRTI	2 (0.86%)
Pneumonia	2 (0.86%)
Cholangitis	1 (0.43%)
COVID	1 (0.43%)
Erysipelas	1 (0.43%)
Meningitis	1 (0.43%)
Pancreatitis	1 (0.43%)
Hematological	26 (11.16%)
ITP	13 (5.58%)
Bernard–Soulier syndrome	3 (1.29%)
SLE	3 (1.29%)
Factor V deficiency	2 (0.86%)
Evans syndrome	1 (0.43%)
Glanzmann’s thrombasthenia	1 (0.43%)
Hyperhomocysteinemia	1 (0.43%)
Red cell aplasia	1 (0.43%)
Sickle cell crisis	1 (0.43%)
Cardiac	15 (6.44%)
PPCM	6 (2.58%)
Adult CHD with heart failure	1 (0.43%)
Atrial dilatation and arrhythmias	1 (0.43%)
HOCM with CCF	1 (0.43%)
Osler-Weber-Rendu syndrome	1 (0.43%)
PAH with RHF	1 (0.43%)
RHD with pulmonary edema	1 (0.43%)
SVT, AF	1 (0.43%)
TOF with CHF	1 (0.43%)
WPW syndrome	1 (0.43%)
Hepatic	12 (5.15%)
Portal HTN	7 (3.00%)
AFLP	5 (2.15%)
Sepsis (Post-partum sepsis)	6 (2.58%)
Respiratory failure	5 (2.15%)
Neurological (Stroke)	2 (0.86%)
Diabetes (Diabetic ketoacidosis)	1 (0.43%)

Table 5 provides details of the categorization of MNM cases based on WHO criteria, including various parameters which fall into clinical, laboratory and management-based criteria of MNM. Among the clinical criteria failure to form clots (n=53, 22.7%) was the common criteria followed by shock (n=31, 13.3%). Among the laboratory-based criteria, acute severe thrombocytopenia was most common (n=78, 33.48%) followed by Oxygen saturation <90% for >60 minutes (n=34, 14.59%). Among the management-based criteria intubation or ventilation not related to anesthesia was most common (n=68, 29.18%), followed by transfusion of >5 units red blood cells (n=53, 22.75%).

**Table 5:** Categorization of MNM based on WHO criteria.

	Criterion	Frequency (%)
Clinical Criteria	Failure to form clots	53 (22.75%)
	Shock	31 (13.30%)
	Respiratory rate > 40 or <6 per minute	29 (12.45%)
	Jaundice	5 (2.15%)
	Oliguria	3 (1.29%)
	Gasping	1 (0.43%)
	Loss of consciousness and absence of PR/ HR	1 (0.43%)
	Cardiac arrest	1 (0.43%)
	Cyanosis	0 (0%)
	Static epilepticus / paralysis	0 (0%)
Laboratory Markers	Acute severe thrombocytopenia	78 (33.48%)
	Oxygen saturation < 90% for > 60 min, PAO2/FiO2 < 200	34 (14.59%)
	Lactate > 5 mEq/mL	25 (10.73%)
	pH < 7.1	20 (8.58%)
	Bilirubin > 6.0 mg/dL	15 (6.44%)
	Creatinine > 3.5 mg/dL	3 (1.29%)
Management-Based Indicators	Intubation/ventilation not related to anesthesia	68 (29.18%)
	Transfusion of > 5 units red blood cells	53 (22.75%)
	Use of continuous vasoactive drugs	46 (19.74%)
	Hysterectomy	44 (18.88%)
	Dialysis for acute renal failure	3 (1.29%)
	Cardiopulmonary resuscitation	3 (1.29%)

Table 6 shows the classification of MNM based on organ dysfunction criteria, with cardiovascular, respiratory, and coagulation dysfunction contributing to the majority of cases. Among the organ-based criteria in cardiovascular dysfunction, the use of continuous vasoactive drugs was the most common (n=46, 19.74%) followed by shock (n=31, 13.3%). Of the respiratory dysfunction criteria, intubation/ventilation not related to anesthesia was the most common (n=68, 29.18%), followed by severe hypoxemia (O2 saturation < 90% for >= 60 min or PFR < 200) (n=34, 14.59%). Of the coagulation dysfunction criteria, severe acute thrombocytopenia was the most common (n=78, 33.48%) followed by equal number of cases of massive transfusion of > 5 units red blood cells (n=53, 22.75%) and failure to form clots (n=53, 22.75%).

**Table 6:** Classification of MNM based on organ dysfunction criteria. MNM: Maternal near miss

	Criterion	MNM (N=233) Frequency (%)
Cardiovascular dysfunction	Shock	31 (13.3%)
	Cardiac arrest	1 (0.43%)
	Use of continuous vasoactive drugs	46 (19.74%)
	Cardiopulmonary resuscitation	3 (1.29%)
	Severe hypoperfusion (Lactate >5 mmol/mL or >45 mg/dL)	25 (10.73%)
	Severe acidosis (pH < 7.1)	20 (8.58%)
Respiratory dysfunction	Acute cyanosis	0 (0.00%)
	Gasping	1 (0.43%)
	Severe tachypnoea/ bradypnea (Respiratory rate > 40 or <6 bpm)	29 (12.45%)
	Severe hypoxemia (O2 saturation < 90% for >= 60 min or PFR < 200)	34 (14.59%)
	Intubation/ventilation not related to anesthesia	68 (29.18%)
Renal dysfunction	Oliguria not responsive to fluids or diuretics	3 (1.29%)
	Dialysis for acute renal failure	3 (1.29%)
	Severe acute azotemia (Creatinine > 3.5 mg/dL)	3 (1.29%)
Coagulation dysfunction	Failure to form clots	53 (22.75%)
	Massive transfusion of > 5 units red blood cells	53 (22.75%)
	Severe acute thrombocytopenia (<50,000 platelets/mL)	78 (33.48%)
Hepatic dysfunction	Jaundice in presence of preeclampsia	5 (2.15%)
	Bilirubin > 6.0 mg/dL	15 (6.44%)
Neurological dysfunction	Metabolic coma (loss of consciousness and presence of glucose and ketoacids in urine)	0 (0.00%)
	Static epilepticus / paralysis	0 (0.00%)
	Coma/Loss of consciousness lasting 12 hours or more	0 (0.00%)

**Figure 1:**
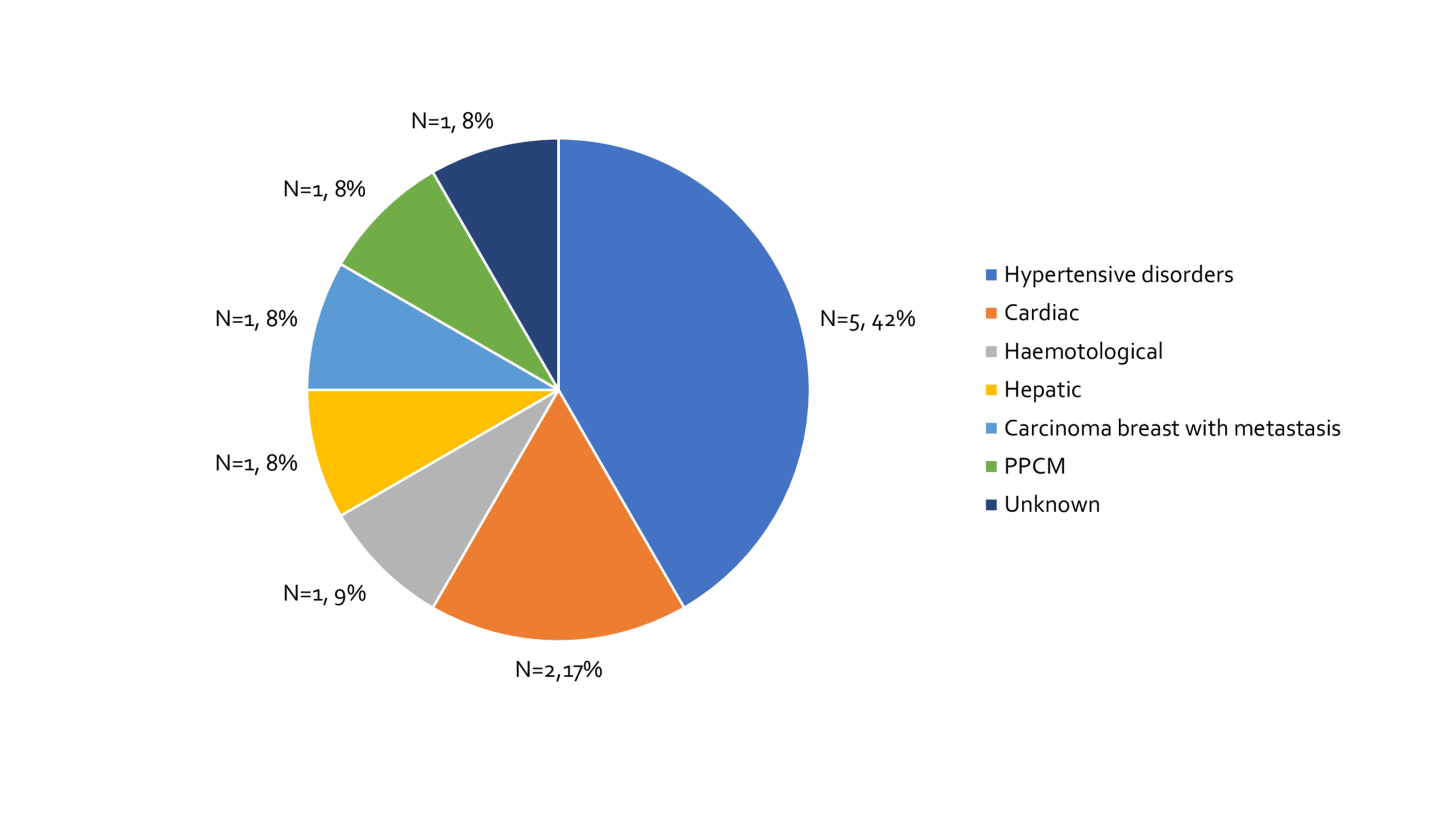
Etiology of maternal deaths. PPCM: Peripartum cardiomyopathy.

There were 12 deaths in the study period, hypertensive disorders accounted for five out of 12 maternal deaths (41.7%) as shown in Figure 1.

## Discussion

Out of the 79,069 women included during the study period, 9.37% had PLTC and 0.31% had LTC. The incidence of MNM (2.95 per 1,000 live births) was much lower in this study when compared to others (7.46-14.34 per 1,000 live births) [15-20]. This difference may be attributed to increased delay in seeking care, women referred late while already in critical condition, low socio-economic status, and lack of resources in primary care centers (e.g., blood banks and round-the-clock availability of skillful personnel) in these studies.

The current study reported a lower MNM than the pooled estimate reported in a recent systemic review carried out by Kulkarni et al. (3.9-379.5 per 1,000 live births). In the systematic review, 25 prospective observational studies from various government health facilities in India were considered. However, there was a wide variation in data because of the different methodologies used in the studies (e.g., study design, study setting, and duration of data collection). Furthermore, the criteria used for defining MNM were different in studies before 2011 [21]. In contrast, in the present study, the same MNM criteria and methodology were used for all 79,069 included cases.

A similar difference in MNM was found in a prospective audit conducted by Manandhar et al. from Kathmandu Medical College. In their study, which lasted one year and included 2,747 births, MNM was 12.5 per 1,000 live births, and the MNM:MD ratio was 17:1. Both of these values are lower than their national averages, as the study setting was in a fee-paying metropolitan area, which contrasts with the majority national demography [22].

In a prospective cross-sectional study conducted by Yemane et al. in Ethiopia, MNM was 24.85 per 1,000 live births. The study was conducted across three tertiary care hospitals for a period of four months, which included 5,530 births. These results are comparable to those from most of the countries of sub-Saharan Africa [23].

Although the above-mentioned studies report differences in morbidity and mortality indices, the etiological factors of MNM are similar (e.g., postpartum hemorrhage, severe preeclampsia, and sepsis) [23]. Gupta et al. reported hemorrhage (40.5%), preeclampsia/eclampsia (24.3%), and sepsis (13.5%) as the major etiological factors responsible for MNM in a year-long study that included 4,786 births, with 74 cases of MNM and 15 cases of MDs. Their (Gupta et al.) study site is a tertiary referral center catering to remote areas where most of the women are referred in a moribund state. In contrast, our study included only women registered for antenatal care early in pregnancy. Although the study by Gupta et al. showed high MNM rates, the etiological factors for MNM were similar to our study [24].

In the last five years, several studies on MNM criteria carried out in various parts of India reported preeclampsia and hemorrhage as the predominant etiological factors, in agreement with the findings of the current study [15-19]. This similarly highlights how etiological factors of MNM are similar across demographic variables.

The maternal morbidity and mortality indices we report for the last 10 years represent the quality of maternal health care services at the study hospital. Similar to the current study, Balachandran et al. reported failure to form clots (24.5%) as the major clinical criterion, and acute severe thrombocytopenia (26.8%) as the major laboratory-based criterion [25].

MI in our study population (4.9%) was lower than that reported by Kumari et al. (34%), whose study included 31,925 births at a tertiary referral center catering to lower socio-economic classes over a study period of 14 months, which may explain this difference [19]. The MNM in the current study is approximately similar to the 5.7% reported by Ingole et al. [20]. Studies by Singh et al. (MI=19.9%, MNM:MD=4:1) and Verma et al. (MNM:MD=2.34:1) agree with our study findings [26,27]. In a prospective study carried out by Prasad et al., MNM:MD was 4.3:1, and MI was 19% [28]. In all of these studies, the population and the study site were comparable to ours, which indicates better obstetric care in the given institutions.

Strengths of the study

The strength of the study is that it includes real-world, high-volume Indian maternity data across 10 years. The study was done in a center that follows a protocol-based approach and has a multidisciplinary teamwork.

The study included women who were registered for care at the study site to look for MNM and MM in these women, reflecting the quality of care at the study site. Identifying hemorrhage and hypertensive disorders as leading causes helps inform local and possibly regional health strategies.

Limitations of the study

This study did have some limitations. Firstly, the study site was a single-specialty center from where women with multiorgan dysfunction may be transferred to a multispecialty center. In addition, the study population did not include women referred in critical condition, which may have affected the final results. The study design is retrospective, and the need for future prospective studies to evaluate the effectiveness of proposed strategies is needed.

## Conclusions

Hemorrhage and hypertension remain the leading causes of MNM and MD. Given the predominance of hemorrhage and hypertensive disorders, early identification protocols, timely referrals, and facility readiness must be prioritized to prevent maternal near-miss events and avoidable deaths. We suggest that every tertiary care center implement strategies to reduce maternal morbidity and mortality. We also strongly recommended that institutes and policymakers bring about necessary regulations for improving maternal health care in India.
